# Computational Study on the Biomechanics of Pupil Block Phenomenon

**DOI:** 10.1155/2019/4820167

**Published:** 2019-09-25

**Authors:** Wenjia Wang, Hongfang Song, Zhicheng Liu

**Affiliations:** ^1^School of Biomedical Engineering, Capital Medical University, Beijing, China; ^2^Beijing Key Laboratory of Fundamental Research on Biomechanics in Clinical Application, Beijing, China; ^3^Beijing Tiantan Hospital, Capital Medical University, Beijing, China

## Abstract

Pupil blocking force (PBF) can indicate the potential risk of pupil block (PB), which is considered as a main pathogenic factor of primary angle-closure glaucoma (PACG). However, the effect of PB on the PBF under different pupil diameters and iris-lens channel (ILC) distance was unknown. Besides, a simple and practical method to assess PBF has not been reported yet. In this study, 21 finite element models of eyes with various pupil diameters (2.4 mm–2.6 mm) and ILC (2 *μ*m–20 *μ*m) were constructed and were conducted to simulate aqueous humor flow by fluid-solid coupling numerical simulation. PBF in each model was calculated based on the numerical simulation results and was fitted using response surface methodology. The results demonstrated that ILC distance had a more significant effect than pupil diameter on PBF. With the decrease of ILC distance, the PBF increased exponentially. When the reduced distance was lower than 5 *μ*m, the PBF exploded quickly, resulting in a high risk of iris bomb. The PBF also varied with pupil diameter, especially under the condition of narrow ILC. Both ILC distance and pupil diameter could explain more than 97% variation in PBF, and a second-order empirical model has been developed to be a good predictor of PBF. Based on the linear relationship between anterior chamber deformation and PBF, a threshold value of PBF was given to guide clinical decisions. This study could be used to investigate PACG pathological correlation and its pathogenesis, so as to provide a reference value for clinical diagnosis of PACG.

## 1. Introduction

Glaucoma is the leading cause of incurable blindness worldwide [1–5]. The most common clinical type in East Asia is primary angle-closure glaucoma (PACG) [6–8], which is characterized by a narrow anterior chamber angle (ACA) [9]. ACA is considered as the outlet of aqueous humor (AH) in the eye. Once the narrowing of ACA occurred, the outflow of AH would be blocked, resulting in an obvious increase in intraocular pressure (IOP), which could cause progressive damage to the structure and function of the optic nerve [10]. Therefore, the maintenance of IOP is critical to the stability of eyeball structure and eye health [11, 12].

**Figure 1:**
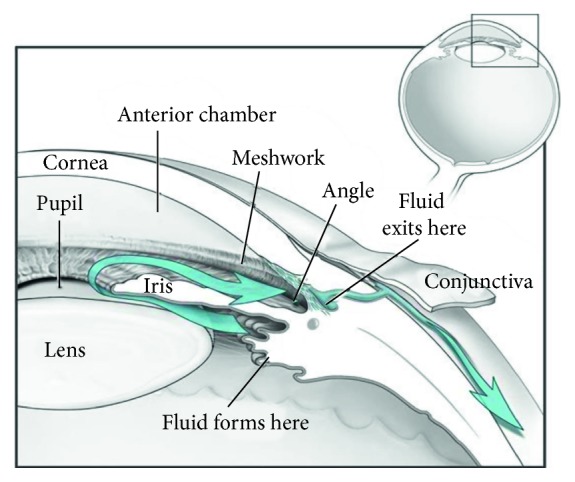
Diagram of the anterior segment of the eye (courtesy: National Eye Institute, National Institutes of Health, Bethesda, MD).

IOP is regulated by the flow of aqueous humor (AH) [13]. AH is secreted by the ciliary body continuously, flows from the posterior chamber (PC) into the anterior chamber (AC) through the pupil, and drains through the trabecular meshwork (Figure 1). Under the normal physiological condition, the rate of AH production is equal to that of drainage, maintaining a stable IOP in the eye. However, many factors can block AH flowing and the most common one is the pupillary block (PB) in PACG patients [14]. Under PB physiological condition, iris-lens channel (ILC) became narrow, which increased the resistance in the flow pathway from PC to AC and hence resulted in a higher IOP in PC and consequently increased the pressure difference between PC and AC [15]. Finally, the elevation of the differential pressure (*P*_d_) would push iris anteriorly and further narrow ACA, which eventually caused a high IOP and further an irreversible damage to the optic nerve.

To quantify the effect of PB on the resistance of AH flow, pupil blocking force (PBF) has been proposed to represent that AH encountered finite resistance in passing through the narrow ILC. By assuming that iris was a type of linear elastic material, a formula based on Hooke's law to calculate PBF has been deduced in early research [16, 17]. However, this method only included the limited physiological influencing factors of PBF and neglected the main resistance in ILC, i.e., AH flow resistance. A method based on computational fluid dynamics (CFD) has been successfully used to study AH flow under PB condition and visualize the flow field [18]. CFD is a powerful tool to analyze the biofluid flow as well as mechanical distribution, and it has been widely used in biomechanics. Therefore, PBF could be assessed precisely by using CFD technology.

As a biomechanical phenomenon, PBF could be affected by a variety of factors. Wang et al. compared the PB degree under different lens' relative position and revealed that lens' position could affect PBF significantly [19]. Huang and Barocas found that lens curvature also had an obvious effect on PBF by setting an active sphincter to simulate PB conditions [9]. Besides, other factors such as AH flow through the iris-lens passage [20, 21] and lens size [22] were proved to be the effect factors of PBF. However, only one factor was investigated in most of the previous research, and a systematic study of various factors has not been reported yet. Moreover, despite high importance, a credible calculation method of PBF was still unknown. Therefore, it was necessary to investigate the comprehensive effect of several factors including pupil diameter, eye size, AC depth, lens size, and its location on PBF as well as AH flow and further construct a computational method to evaluate PBF.

The aim of this study was to investigate the effect of different optical conditions on PBF and construct a model to evaluate PBF. Firstly, twenty-one eye models with different pupil diameters and ILC were constructed and were conducted with numerical simulations. Then, PBF was calculated based on a modified Bernoulli's equation. After that, response surface methodology was utilized to fit the relationship between PBF and pupil diameter as well as ILC distance. The effect of pupil diameter, ILC distance, and their interaction on PBF was also discussed. Finally, the AC morphology including variation in ACA and iris deformation under different PBF was investigated and a threshold value of PBF was given to guide clinical decisions.

## 2. Methods

### 2.1. Pupil Blocking Force

**Figure 2:**
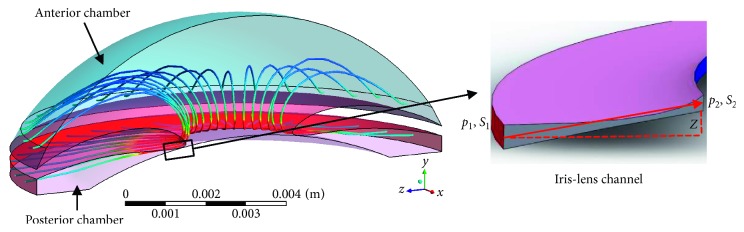
The trajectories of AH flow from PC to AC and its flow through ILC (enlarged figure).

AH mainly flows from PC to AC through ILC. Assuming that AH was ideal fluid, its mechanical energy should remain constant at any position based on Bernoulli's equation. However, due to complex components, the viscidity of AH could not be neglected [23]. Considering the fluid viscosity, Bernoulli's equation could be modified as

(1) $$\begin{array}{c} p+\frac{1}{2}\rho v^{2}+\rho gh+h_{\text{f}}=C, \end{array}$$

where *p* is the pressure at one position, *h* is its height, *ρ* is the fluid density, *v* is the fluid velocity, *g* is the acceleration of gravity, *h*_f_ represents the flow resistance during AH flow pathway, and *C* is constant. When AH flows through ILC (Figure 2), the following equation could be induced to describe the conservation of energy:

(2) $$\begin{array}{c} 0+\frac{1}{2}u_{1}^{2}+\frac{p_{1}}{\rho }=Zg+\frac{1}{2}u_{2}^{2}+\frac{p_{2}}{\rho }+H_{\text{f}}, \end{array}$$

where *u*_1_ and *u*_2_ are the average velocities in the two surfaces (*S*_1_ and *S*_2_), *p*_1_ and *p*_2_ are the pressures in these surfaces, *A*_1_ and *A*_2_ are their areas, respectively, *Z* is the vertical distance from *S*_1_ to *S*_2_, and *H*_f_ (unit of J/kg) is the resistance (i.e., PBF) when AH flows from *S*_1_ to *S*_2_ [14, 24].

Known that AH is secreted by the ciliary body, its volumetric flux (represented by *Q*) in the surfaces *S*_1_ and *S*_2_ is identical and can be calculated as the multiplication by area and velocity; therefore, the following equation can be obtained:

(3) $$\begin{array}{c} Q=S_{1}\cdot u_{1}=S_{2}\cdot u_{2}. \end{array}$$

By applying equation (3), equation (2) can be written as equation (4) and PBF can be calculated based on the results of pressure and area:

(4) $$\begin{array}{c} H_{\text{f}}=\frac{p_{1}-p_{2}}{\rho }+\frac{Q^{2}}{2}\left(A_{1}^{-2}-A_{2}^{-2}\right)-Zg. \end{array}$$

### 2.2. Geometrical Model

**Figure 3:**
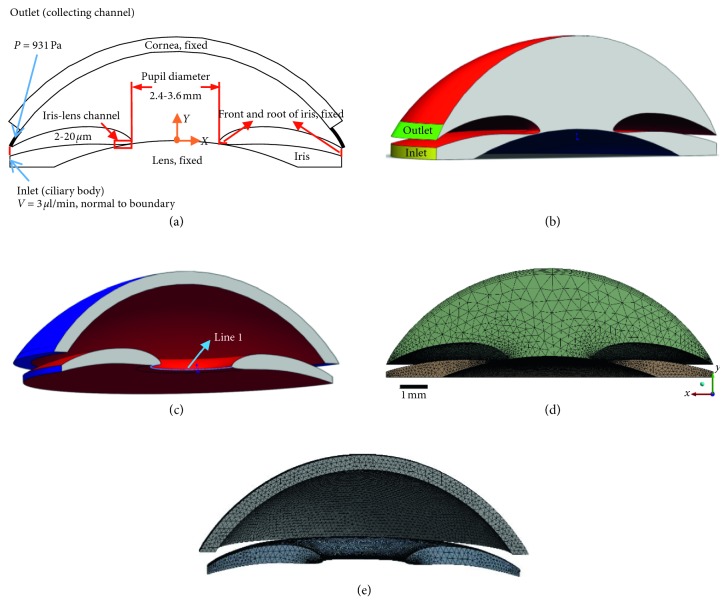
Schematic diagram of the finite element model and its computational grid: (a) 2D scheme of the model; (b) 3D scheme of AH; (c) 3D scheme of iris and cornea; (d) computational grids of AH; (e) computational grids of iris and cornea.

Due to the hard-to-measure pressure and area in real eye, numerical simulation is used to simulate the AH flow and assess those parameters in equation (4). The geometric models for finite element analysis, including cornea, iris, lens, and AH (Figures 3(a)–3(c)), were developed from the finite model in our previous study [18]. All the models were established with similar geometry parameters as typical ocular dimensions [25–27], with the assumption that the anterior segment was axisymmetric. Notably, different ILC distances as well as pupil diameters were used to construct these models, while the other geometric parameters remained the same. Detailed dimensions of the models are shown in Table 1. Considering the fact that obvious resistance in AH could be observed when the ILC distance was under 20 *μ*m and PB was defined when the distance was less than 5 *μ*m [28, 29], seven levels (20 *μ*m, 15 *μ*m, 10 *μ*m, 5 *μ*m, 4 *μ*m, 3 *μ*m, and 2 *μ*m) of ILC distances were chosen to simulate healthy and different PB conditions. Three levels (2.4 mm, 3 mm, and 3.6 mm) of pupil diameters [30] were set to simulate different pupil sizes in PACG patients. Based on full factorial design, twenty-one models were constructed and are shown in Figures 3(a)–3(c). All of these models were conducted to generate the tetrahedron mesh using ICEM CFD and Mechanical APDL (Figures 3(d) and 3(e)). The mesh was refined, and the independency was checked. The computational grids were exported to ANSYS Workbench (ANSYS Inc., Canonsburg, PA, USA) for fluid-solid coupling simulation analysis.

### 2.3. Fluid-Solid Coupling Analysis

In order to calculate the *P*_d_ between surfaces *S*_1-2_ and investigate the effect of PB on eye shape with different pupil diameters, all the finite element models were conducted to simulate AH flow by fluid-solid coupling numerical simulation. To model the motion of AH steady flow from PC to AC, the Navier–Stokes equation representing the conservation of the momentum is written as

(5) $$\begin{array}{c} \rho \left(u\cdot \nabla u\right)=-\nabla p+\mu \nabla^{2}u+\rho g. \end{array}$$

From left to right, the terms in equation (5) represent the change in momentum caused by convection, pressure gradients, viscous diffusion, and gravity. Considering AH is an incompressible Newtonian viscous fluid, the continuity equation is given as

(6) $$\begin{array}{c} \nabla \cdot u=0. \end{array}$$

For the boundary conditions, the inlet (the yellow surface in Figure 3(b)) was considered as constant flux and the flux rate was set at 3 *μ*L/min, which was equal to the generation rate of AH in real eyes [32]. The outlet (the green surface in Figure 3(b)) was set as constant pressure (scleral venous pressure) for AH fluid. The exterior surface of lens, iris root (the blue surfaces in Figure 3(c)), and pupil (Figure 3(c) line 1) were set as fixed positions. The interface between the cornea and AH and the interface between iris and AH (the red surfaces in Figures 3(b) and 3(c)) were chosen as fluid-solid interaction surfaces to simulate the interaction between AH fluid and solid bodies [25]. Moreover, the bottom (the blue surface in Figure 3(b)) was applied to the wall boundary condition. As for the material properties, the liquid properties in the simulation were assumed to be those of pure water. The solid properties of cornea and iris were set according to the measurement of real eyes. A nonlinear elasticity model (2^nd^ Ogden model) was used to simulate the mechanical properties of iris. Cornea was considered to be a linear elastic material and iris was a nonlinear elastic material. Detailed information about material properties is shown in Table 2.

### 2.4. Soft Measurement Method of PBF

Based on the result of fluid-solid coupling analysis, the *H*_*f*_ in each model was calculated by equation (4). In order to investigate the effect of pupil diameters, ILC distances, and their interaction on PBF, the response surface methodology (RSM) was selected to fit the experimental data. RSM was considered as a simple and fast empirical tool to study both the effect of each individual factor and their interactions on the response variable [38, 39]. By using SPSS Statistics software, a RSM model was constructed and was optimized with a sequential quadratic programming approach. After that, analysis of variance was used to verify the significance of the optimized model and its parameters. Finally, an empirical equation was derived to predict PBF based on the measurement of ILC distance and pupil diameter.

### 2.5. Analysis and Calculation

The areas of surfaces *S*_1-2_ (*A*_1-2_) were calculated using SolidWorks2014 software, and the pressures (*P*_1-2_) in those surfaces were derived from the results of fluid-solid coupling analysis by CFD-Post in ANSYS Workbench. Then, the magnitude of PBF was calculated by equation (4) according to the above parameters. The deformation of iris (De), equivalent stress, and equivalent elastic strain of iris were also obtained by CFD-Post. Because of the irregular curved surface of the iris, the anterior chamber angle can be measured by averaging the ACA values in the results of iris deformation images of repeated manual measurements.

## 3. Results and Discussion

### 3.1. Effects of Iris-Lens Channel Distance and Pupil Diameter on AH Flow

**Figure 4:**
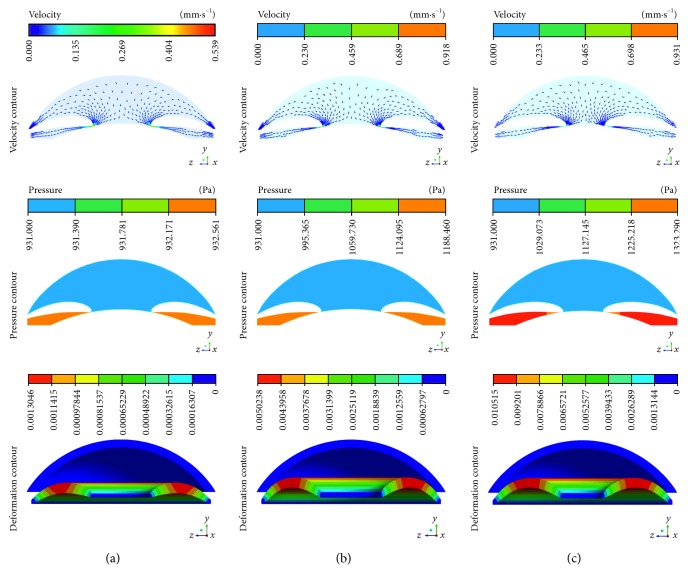
Contours of the velocity and pressure with various ILC distances and pupil diameters: (a) 20 *μ*m and 3.6 mm; (b) 2 *μ*m and 3.6 mm; (c) 2 *μ*m and 2.4 mm.

With various ILC distances and pupil diameters, the velocity and pressure distributions of AH within eyes are shown in Figure 4, where gravity was opposite to the direction of *y*-axis, representing the supine position in clinical. It was obvious that the main flux of AH from PC to AC was at a low velocity. However, the fluid was accelerated rapidly and the velocity achieved peak when AH flowed through ILC. Besides, an obvious difference in pressure between PC and AC could be observed because of the resistance of ILC, according to a previous study [40]. The maximum velocity of AH (*V*_max_) and the *P*_d_ between PC and AC were different with various ILC distances and pupil diameters. Despite the same pupil diameter (3.6 mm), the *V*_max_ and *P*_d_ were 5.39*e* − 4 m/s and 1.60 Pa when the ILC distance was 20 *μ*m (Figure 4(a)), while they became 1.72*e* − 3 m/s and 257.00 Pa once the distance decreased to 2 *μ*m (Figure 4(b)). When the pupil diameter decreased from 3.6 mm to 2.4 mm, the *V*_max_ and *P*_d_ increased to 2.13*e* − 3 m/s and 392.00 Pa further, indicating both the ILC distance and diameter had effects on the *V*_max_ as well as *P*_d_. The previous study has demonstrated that *V*_max_ and *P*_d_ could influence AC morphology [41], which was also confirmed by the AC deformation in our study (Figure 4). Iris bomb and ACA blocking could be observed obviously when the ILC became narrow (Figures 4(a) and 4(b)), according to clinical trials [42]. Notably, the degree of iris bomb and ACA blocking varied with pupil diameter, indicating that pupil diameter was also an important factor to assess the risk of PB [43].

**Figure 5:**
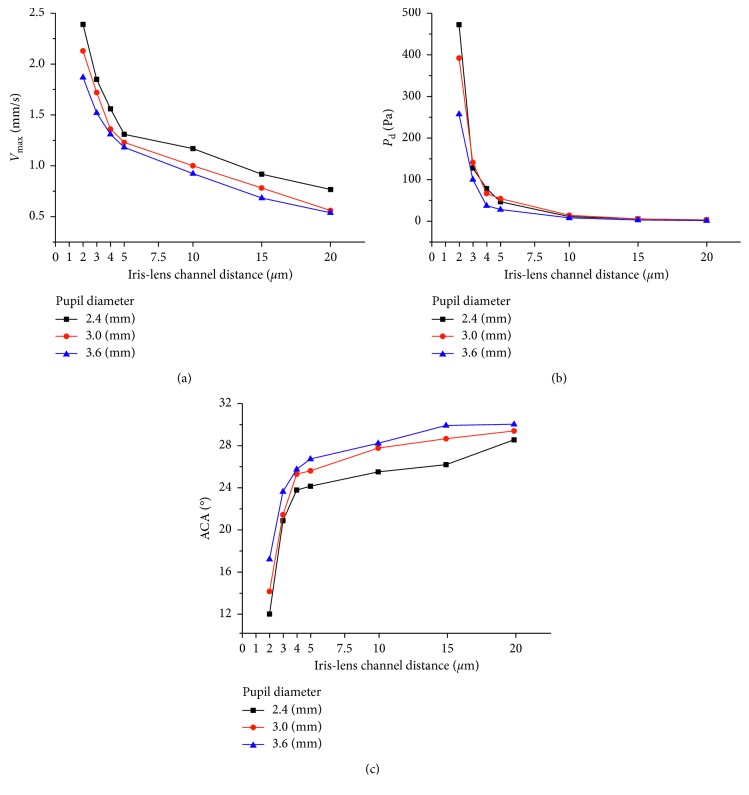
The relationship between *V*_max_ and ILC distance under various pupil diameter levels (a), *P*_d_ and ILC distance under various pupil diameter levels (b), and ACA and ILC distance under various pupil diameter levels (c).

The results of *V*_max_ and *P*_d_ under different ILC distances and pupil diameters are shown in Figure 5. With the decrease of ILC distance, an exponential increase of *V*_max_ and *P*_d_ could be observed under each level of pupil diameter (Figure 5(a)). Besides, both *V*_max_ and *P*_d_ varied with pupil diameter, especially under narrow ILC distances (Figures 5(a) and 5(b)). Similarly, an exponential increase of ACA could be observed with the decrease of ILC distance (Figure 5(c)), indicating a great effect of PB on AC morphology. The effect of pupil diameter on iris deformation showed greater differences under different levels of ILC distances (Figure 5(c)). When the pupil diameter decreased from 3.6 mm to 2.4 mm, ACA increased 85.48% under the ILC distance of 2 *μ*m while only 23.88% under the ILC distance of 20 *μ*m. Furthermore, the curves of the relationships between *V*_max_ and ILC distance were not parallel under each level of pupil diameter. Similar phenomena could be observed in Figures 5(b) and 5(c), suggesting a potential interaction between ILC and pupil diameter on *V*_max_, *P*_d_, and iris deformation [44].

### 3.2. Soft Measurement Method of PBF

**Figure 6:**
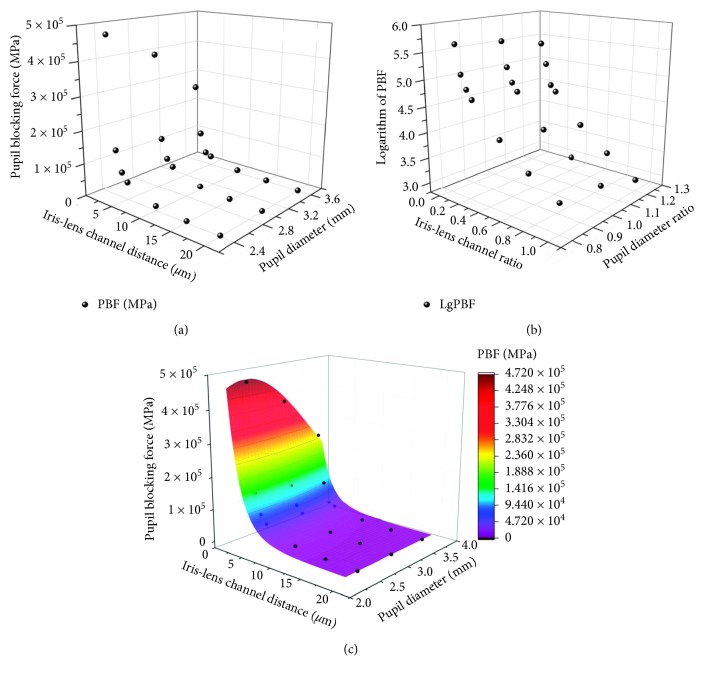
The experimental data and predicted model of PBF with various ILC distances and pupil diameters: (a) the relationship between PBF and ILC distance as well as pupil diameter; (b) the relationship between logarithmic PBF and ILC ratio as well as diameter ratio; (c) the 3D response surface plot and experimental data.

Considering the obvious effects of ILC as well as pupil diameter on the *P*_d_ and AH flow velocity, it could be induced that these two factors also had an impact on PBF. The results of PBF in the twenty-one models are shown in Figure 6(a). A distinct difference in PBF values could be observed under various ILC distances. It was only 1049.2 J/kg − 2342.4 J/kg under a wide ILC (20 *μ*m) while it achieved 256449.2 J/kg − 472235.6 J/kg under an extreme narrow ILC (2 *μ*m). This phenomenon verified the rationality of ILC as an indicator to assess PB risks. Moreover, similar trends could be observed in the relationship between the ILC distance and PBF under each level of pupil diameter, which appeared to be exponential growth curves. The PBF exploded when the ILC distance decreased to less than 5 *μ*m, verifying the reliability of 5 *μ*m as the threshold of PB in clinical [28, 29]. Furthermore, the PBF values increased in different degrees under various ILC distances with the same change of pupil diameter from 3.6 mm to 2.4 mm, showing a potential interaction relationship between ILC distance and pupil diameter on PBF.

Despite high accuracy to assess PBF using finite element analysis, it was not suitable for clinical application because of complex operation and high cost. Therefore, it was necessary to establish a soft measurement method to evaluate PBF. Considering the exponential distributions of PBF under each pupil diameter level, the logarithm of PBF value was used to develop a regression model. Besides, ILC ratio and diameter ratio, defined by the ratio of the measured ILC distance and pupil diameter to normal values (ILC distance: 20 *μ*m; pupil diameter: 3 mm), were calculated to eliminate the discrepancy in magnitude. The relationships between logarithmic PBF and the ILC ratio as well as the diameter ratio are shown in Figure 6(b). By applying multiple regression analysis on these data, a second-order polynomial equation for the logarithmic PBF was obtained as follows:

(7) $$\begin{array}{c} y=2.328x_{1}^{2}-1.749x_{2}^{2}-0.263x_{1}x_{2}-4.573x_{1}+2.910x_{2}+4.682, \end{array}$$

where *y* represents the logarithmic PBF and *x*_1_ and *x*_2_ are the ILC ratio and pupil diameter ratio, respectively. The result of variance analysis showed that the determination coefficient of this model achieved 0.973, implying approximately 98% of the variability in logarithmic PBF could be expressed by the model. Therefore, the empirical relationship between PBF value and the measured parameters (including ILC distance and pupil diameter) was quantified using the following equation:

(8) $$\begin{array}{c} Y=48083.935\cdot 10^{\left(0.00596X_{1}-0.194X_{2}-0.00438X_{1}X_{2}-0.229X_{1}+0.970X_{2}\right)}, \end{array}$$

where *Y* represents the value of PBF (J/kg) and *X*_1_ and *X*_2_ are, respectively, the measured ILC distance (*μ*m) and pupil diameter (mm).

### 3.3. Effect of PBF on AC Morphology

**Figure 7:**
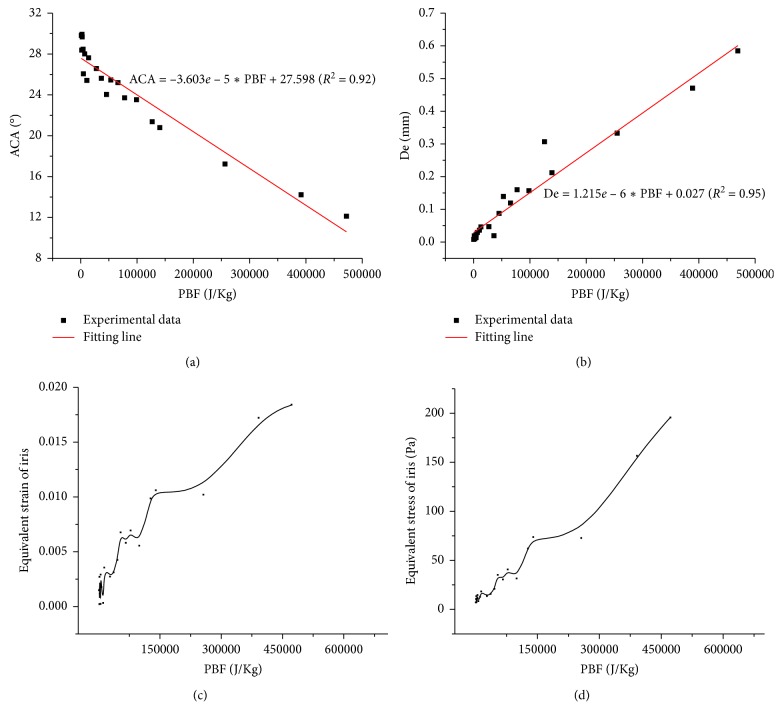
The relationships and fitting lines between ACA and PBF (a) and De and PBF (b); the relationships between equivalent elastic strain and PBF (c) and equivalent elastic stress and PBF (d).

In order to testify the feasibility of PBF as an indicator to assess the risks of PB in clinical, the relationship between PBF and variation of ACA was investigated and is shown in Figure 7. ACA increased with PBF, and a linear relationship was observed between ACA and PBF. The determination coefficient achieved 0.92, indicating that more than 92% variation in ACA could be explained by PBF. Similar phenomenon could be observed in the relationship between deformation of iris and PBF. With the increase of PBF, the deformation of iris increased linearly. The determination coefficient of the fitted line was 0.95, suggesting the linear model was able to express more than 95% of the variability in iris deformation. Iris deformation and variation in ACA were considered as the pathogenic mechanism of PACG [45]. Increased iris deformation could decrease AC depth and narrow ACA, resulting in a severe obstruction of AH outflow, which could cause high IOP in eye and induce acute attack of angle-closure glaucoma. Therefore, PBF could be utilized as an indicator to assess the effect of PB on AC morphology and evaluate its risks. Furthermore, obvious tendency of increase in equivalent elastic strain and equivalent stress of iris could be observed with the increase of PBF. The equivalent elastic strain of iris represented its deformability, which could be utilized to evaluate the risk of iris bomb. Increased equivalent stress of iris under high PBF could hasten the detachment of epithelial cells in iris, which might cause an obstruction in AC and rise the resistance of AH outflow [46], verifying the reliability of PBF to assess the risks of BP.

According to clinical experience, obvious deformation of iris and narrowing of ACA could be observed when ILC distance decreased to 5 *μ*m with a 3 mm pupil diameter. The value of PBF under that pathology condition was 45835.6 J/kg. Considering the effect of pupil diameter on PBF, the value should fluctuate in the range from 27849.2 J/kg to 53843.4 J/kg. As mentioned in Section 3.2, a quadratic polynomial equation has been solved to explain the variation in PBF, and a second-order empirical model has been developed to provide a good estimate of PBF. Based on the linear phenomena between PBF and ACA as well as PBF and De, it was reasonable to use the PBF value of 27000 J/kg as PB risk threshold in clinical. Further study will focus on the optimization of the empirical model of PBF to enlarge the range of pupil diameter.

## 4. Conclusions

This study presented a simple and practical method to assess PBF by the measurement of ILC distance and pupil diameter, which were accessible parameters in clinical. The results demonstrated that the ILC distance had an obvious effect on PBF. In a specific range of pupil diameter from 2.4 mm to 3.6 mm, the ACA became narrow with the decrease of the diameter. The quantitative analysis of PBF in this study could be used to research PACG pathological correlation and its pathogenesis, so as to provide a threshold for clinical diagnosis of PACG.

## Figures and Tables

**Table 1:** The geometrical parameter of finite element models.

Quantity	Finite element model	Sources
Diameter of the anterior chamber	13 mm	[31]
Maximum height of the chamber	2.63 mm	[31]
Maximum radius of curvature of the posterior cornea	6.8 mm	[31]
Radius of curvature of the natural lens	10 mm	[31]
Height of the iris-lens channel	2–20 *μ*m	[14, 31]
Diameter of pupil	2.4–3.6 mm	[30]
Angle between cornea and iris	30°	[31]

**Table 2:** Material properties of AH, cornea, and iris in finite element models.

Material properties	Value	Sources
AH density	1000 kg·m^−3^	[33]
AH viscosity	0.001 kg^−1^·s^−1^	[34]
AH volumetric flux secreted by ciliary body, *V*	3 *μ*L/min	[32]
AH outlet pressure (scleral venous pressure), *P*	7 mmHg	[35]
Cornea density	1143 kg·m^−3^	[36]
Cornea Young's modulus	1.5 MPa	[36]
Iris density	1000	[37]
Iris 2^nd^ Ogden material coefficients	*μ*_1_ = 43.05 kPa, *μ*_2_ = 37.7 kPa	[37]
	*α*_1_ = 54.255, *α*_2_ = 48.072	

## Data Availability

The data used to support the findings of this study are available from the corresponding author upon request.
